# Who will increase their physical activity? Predictors of change in objectively measured physical activity over 12 months in the *ProActive *cohort

**DOI:** 10.1186/1471-2458-10-226

**Published:** 2010-04-30

**Authors:** Rebecca K Simmons, Esther MF van Sluijs, Wendy Hardeman, Stephen Sutton, Simon J Griffin, the ProActive project team

**Affiliations:** 1 MRC Epidemiology Unit, Institute of Metabolic Science, Box 285, Addenbrooke's Hospital, Hills Road, Cambridge, CB2 0QQ, UK; 2 General Practice & Primary Care Research Unit, Institute of Public Health, Forvie Site, Robinson Way, Cambridge, CB2 0SR, UK

## Abstract

**Background:**

The aim was to identify predictors of change in objectively measured physical activity over 12 months in the *ProActive *cohort to improve understanding of factors influencing change in physical activity.

**Methods:**

*ProActive *is a physical activity promotion trial that took place in Eastern England (1999-2004). 365 offspring of people with type 2 diabetes underwent measurement of physical activity energy expenditure (PAEE) using heart rate monitoring, fitness, and anthropometric and biochemical status at baseline and 1 year (*n *= 321). Linear regression was used to quantify the associations between baseline demographic, clinical, psychosocial and behavioural variables and change in PAEE over 12 months. This study is registered as **ISRCTN**61323766.

**Results:**

*ProActive *participants significantly increased their PAEE by 0.6 kj/min (SD 4.2, p = 0.006) over one year, the equivalent of around 20 minutes brisk walking/day. Male sex and higher fitness at baseline predicted increase in PAEE. No significant associations were found for any other variables. Very few baseline demographic, clinical, psychosocial and behavioural predictors were associated with change in objectively measured physical activity.

**Conclusions:**

Traditional baseline determinants of self-reported physical activity targeted by behavioural interventions may be relatively weak predictors of change in objectively measured physical activity. Further research is needed to improve our understanding of factors influencing change in physical activity to inform the development and targeting of interventions.

## Background

Physical activity levels have been shown to predict risk of type 2 diabetes [1], cardiovascular disease [2], osteoporosis [3], some forms of cancers [4] and depression [5]. Encouraging people to increase their activity levels is therefore high on the public health agenda of most developed nations. Physical activity promotion interventions typically target individuals on the basis of risk according to one or more factors, including age, sex, current physical activity status, family history of disease, smoking, overweight or socio-economic status [6-12]. Results from trials suggest that these interventions work for some individuals but not for others. It is often those at highest risk who show the least change e.g. sedentary or disadvantaged individuals, smokers, and the elderly [7,8,13,14]. If it were possible to identify those who are most likely to respond positively to particular health promotion programmes, health resources could be allocated to appropriate sub-groups, who are either more or less likely to change. Selection of target groups and development of interventions could thus be enhanced by a better understanding of the predictors of change in physical activity among relevant populations.

Most studies exploring predictors of physical activity behaviour change measure *current or future *behaviour, and frequently focus on sporting activities [15]; the majority also rely on *self-reported *physical activity [14,16-18]. Therefore, we aimed to investigate whether selected predictors were associated with change in objectively measured physical activity over 12 months in a health promotion trial cohort (*ProActive*) to inform subsequent intervention development and targeting. As physical activity behaviour is influenced by a variety of factors from multiple domains [19], we included a wide range of baseline predictors. We examined demographic variables, including age, sex, and socio-economic status, that have already been shown to be predictive of physical activity, or change in physical activity [20]. As the behavioural determinants targeted in the intervention were informed by the Theory of Planned Behaviour based on meta-analytic evidence for their ability to predict behaviour [15,21-24], we included variables which have been shown to be positive proximal predictors of physical activity (intention and perceived behavioural control), and included past activity behaviour, which has also been to shown to predict future behaviour [15]. Similarly, BMI was considered as a potential predictor as it has previously been linked to physical activity behaviour change [16,17,25]. We also looked at variables that have not previously been examined in order to generate new hypotheses regarding the prediction of change in physical activity. These included clinical variables, such as blood pressure and cholesterol, and psychosocial variables, such as self-reported anxiety and health status, which may not be causally related to change, but may form part of a pragmatic strategy to identify those who might benefit from changing their physical activity behaviour.

## Methods

*ProActive *is a randomised trial of a theory- and evidence-based behavioural intervention to increase physical activity among individuals who are at increased risk of the consequences of a sedentary lifestyle due to their family history of type 2 diabetes [26]. Potential participants were identified via diabetes registers and medical records of family history in 20 general practices in East Anglia, England.

Participants were initially recruited through their parents who were identified on diabetes registers. 2,631 patients were approached and 2,025 (77%) replied, yielding 1,238 potentially eligible offspring who were invited to take part in the study. In seven of the practices, 1,340 patients with a recorded family history of diabetes were written to, and 896 (67%) responses were received, with 283 patients interested and eligible. Potential participants were asked to fill in a short screening activity questionnaire, describing occupational and leisure activity, based on published questionnaires [27,28], to exclude very active individuals. Out of 1,123 individuals who completed the questionnaire (74% response rate), 323 refused to participate, 411 met the exclusion criteria and 24 took part in a pilot study. Therefore, 365 individuals aged 30-50 years and reporting low levels of activity were randomly assigned to one of three interventions: (i) a theory-based leaflet with brief advice; (ii) the leaflet plus an intensive theory-based behaviour change programme delivered at participants' homes; or (iii) the leaflet plus the same intervention programme delivered by telephone. The programme was delivered by trained facilitators (for further details see [29]). It aimed to support increases in physical activity through a range of self-regulatory skills including goal setting, action planning, self-monitoring, and relapse prevention. Information on a range of demographic, clinical, psychosocial and behavioural variables was collected from participants, as well as objective measurement of physical activity (using heart rate monitoring) at baseline and one year follow-up. Main trial results indicated that the intensive theory-based behavioural intervention was no more effective than the theory-based brief advice leaflet alone in increasing objectively measured physical activity levels at one year [30]. However, all participants increased their physical activity, on average, by the equivalent of 20 minutes brisk walking a day. In order to examine the predictors of increased physical activity, we pooled the trial arms to conduct a cohort analysis. Ethical approval was obtained from the Eastern England MREC (02/5/53) and relevant Primary Care Trusts; all participants gave written informed consent. Full details of the study have been reported elsewhere [26].

### Measurement of physical activity energy expenditure

Participants wore heart rate (HR) monitors (Polar Electro Ltd, Kemple, Finland) continuously during the waking hours over 4 days following their visit to the study centre. Resting energy expenditure (REE) was calculated by the Weir formula [31] using VO_2 _and VCO_2 _measurements obtained in the fasting state, after approximately 10 minutes of supine rest using the same indirect calorimetry system as previously described [26]. Physical activity energy expenditure (PAEE) was measured using the flex HR method, which has been described in detail elsewhere [32,33]. Below the flex HR point, energy expenditure (EE) was assumed to be equivalent to REE. EE above the flex point was predicted from the individual HR-EE regression line. PAEE was calculated by subtracting REE from the estimated average daily energy expenditure, and thereafter averaged over the 4-day period.

### Clinical predictors

Participants attended the study centre after an overnight fast and a sample of venous blood was taken. Total cholesterol was measured using standard enzymatic methods, as previously described [26]. Glycosylated haemoglobin (HbA_1c_) was measured on fresh EDTA blood samples using high-performance liquid chromatography. Weight was measured on standard calibrated scales and height was measured using a rigid stadiometer. BMI was calculated as weight (kg) divided by height squared (m^2^). Systolic and diastolic blood pressure were measured in a seated position using an automated Accutorr sphygmomanometer (Accutorr, Cambridge, UK) three times at one-minute intervals; the mean of these measurements was used in the analysis [26]. Aerobic fitness (VO_2max_) was predicted as oxygen uptake at estimated maximal HR (220 minus age) by extrapolation of the regression line established during the individual calibration for the relationship between oxygen consumption and HR during a submaximal graded treadmill exercise test.

### Psychosocial predictors

Self-report measures of well-being and quality of life were collected, including the SF-36 health survey [34], the short form of Spielberger's State Anxiety Inventory (STAI) [35] and a measure of current health status (EuroQol). In brief, the SF-36 is a health survey of 36 items which can be used to generate psychometrically-based physical and mental health summary measures. The STAI consists of six, 4-point Likert scales, and has been shown to correlate well (r > 0.9) with the results of the longer questionnaire from which it was derived [35], which is in turn associated with indicators of anxiety [36]. The STAI used in this study measures state (current state of anxiety or mood) rather than trait (recurring or individual characteristics of anxiety or mood) anxiety [35]. The EuroQol is a health "thermometer" which asks responders to mark on a scale of 0 to 100 their current health status, from "Worst imaginable health state (0)" to "Best imaginable health state (100)". We also collected data on cognitive predictors of physical activity, based on the Theory of Planned Behaviour, that were hypothesized to be directly associated with behaviour change [29]. Items were developed according to Theory of Planned Behaviour guidelines [37] and measured on a Likert-type scale ranging from 1 (*strongly disagree*) to 5 (*strongly agree*) [38], and averaged to calculate overall scores. The questionnaire included two items to assess perceived behavioural control ('it would be difficult for me to be more physically active in the next 12 months even if I wanted to' (score reversed), 'I am confident that I could be more physically active in the next 12 months, if I wanted to') and intention ('I intend to be more physically active in the next 12 months', 'it is likely that I will be more physically active in the next 12 months'). Cronbach's alpha for internal consistency was 0.54 for perceived behavioural control, and 0.77 for behavioural intention at baseline.

### Behavioural predictors

Past activity behaviour was measured using the EPIC-Norfolk physical activity questionnaire (EPAQ-2), which has previously been validated using objective heart rate monitoring [39]. Participants answered questions about smoking status and weekly alcohol consumption in a general health questionnaire.

### Statistical analyses

Descriptive summary statistics were calculated separately for men and women using means and SDs at baseline and follow-up. T-tests were used to examine whether there were any differences in baseline characteristics between those with and without follow-up data. We used linear regression to model baseline demographic, clinical, behavioural and psychosocial variables separately against PAEE at follow-up. These models were adjusted for baseline PAEE to describe change in this variable over time. We also ran a multivariable regression model to examine PAEE change, mutually adjusting for all significant predictor variables (p < 0.05), to establish which variables were independently associated with the outcome. The residuals of all linear regression models were checked to ensure they were normally distributed. Models were also run separately by trial arm and by sex. As results were similar in the three trial arms and by sex (data not shown), the data were pooled and results from linear regression models conducted in the whole cohort are presented. Type I error was set at 0.05 for all tests. All data were analyzed in continuous form using Stata Version 10.0. (STATA Corp., College Station, Texas, USA).

## Results

Complete data on PAEE, aerobic fitness, anthropometry and biochemical measures were available in 365 participants at baseline and 321 participants at 12 months. Participants with missing data at follow-up were similar to those with complete data on age, sex, and social class, as well as baseline BMI, PAEE and fitness (all p-values > 0.05, data not shown).

Table 1 shows characteristics of participants at baseline and follow-up stratified by sex (n = 321). Men had significantly higher blood pressure and HbA_1c _levels than women at baseline, and reported higher levels of alcohol consumption. Statistically significant differences were also observed for PAEE and VO_2max_, with men having higher levels of PAEE and fitness than women at baseline.

**Table 1:** Characteristics of *ProActive *participants with complete baseline and follow-up data, stratified by sex, *n *= 321

	Men (*N *= 129)		Women (*N *= 192)	
	
	Baseline	Follow-up	Baseline	Follow-up
Age (years)	40.2 (5.8)	-	40.8 (6.1)	-
Social class (based on the Index of Multiple	4.2 (0.9)	-	4.2 (0.9)	-
Deprivation score 1-5; 5 representing highest social class)				

BMI (kg/m^2^)	28.2 (4.3)	28.3 (4.3)	27.6 (5.2)	27.8 (5.4)^a^
Systolic blood pressure (mmHg)	127.1 (11.3)	124.5 (11.3)^a^	120.6 (13.8)^b^	116.8 (13.4)^c^
Diastolic blood pressure (mmHg)	81.4 (8.8)	79.6 (9.7)^a^	76.2 (9.3)^b^	73.9 (9.9)^c^
HbA_1c _(%)	5.3 (0.5)	5.5 (0.7)^c^	5.2 (0.5)^b^	5.3 (0.4)^c^
Total cholesterol (mmol/l)	5.3 (1.0)	5.3 (1.0)	5.1 (0.9)	5.2 (1.0)

Perceived behavioural control (scale 1-5; 5 representing highest perceived control)	3.8 (0.6)	3.6 (0.7)^a^	3.9 (0.6)	3.6 (0.7)^c^
Intention (scale 1-5; 5 representing highest level of intention)	3.7 (0.6)	3.7 (0.7)	3.7 (0.6)	3.6 (0.7)^a^
Current health status (scale 0-100, where 0 = worst imaginable health to 100 = best imaginable health state)	77.8 (14.7)	80.8 (13.6)^a^	79.0 (15.7)	81.7 (12.7)^a^
Spielberger state anxiety score (scale 20-80, where 80 represents the highest possible score)	36.4 (11.6)	33.8 (10.7)^a^	38.6 (12.6)	37.4 (12.0)
SF-36 physical functioning score (scale 0-100, with 100 indicating highest level of self-reported physical health)	90.9 (16.5)	95.5 (7.9)^c^	90.2 (14.2)	93.0 (12.4)^a^
SF-36 mental health score (scale 0-100, with 100 indicating highest level of self-reported mental health)	76.6 (15.0)	79.2 (15.0)^a^	74.0 (14.9)	75.4 (16.7)

Self-reported alcohol (total units/wk)	10.6 (10.6)	N/A	4.6 (5.7)^b^	N/A
Self-reported smoking status, n (%) current smoker	27 (20.9)	25 (19.4)	29 (15.1)	26 (13.5)

PAEE (kj/min)	8.2 (5.0)	9.1 (5.4)	4.6 (3.5)^b^	5.1 (3.2)^a^
VO_2max _(l/min)	4.0 (0.9)	4.0 (0.9)	2.7 (0.6)^b^	2.8 (0.6)

Data are means (SD), except where stated otherwise. Means were compared using paired t-tests for baseline vs follow-up, and t-tests for men vs women at baseline. Proportions were compared using McNemar's test for baseline vs follow-up and a chi-squared test for men vs women at baseline. N/A = not available; ^a ^p < 0.05 for baseline vs follow-up (separately in men and women); ^b ^*p *< 0.001 for women vs. men at baseline; ^c ^p < 0.001 for baseline vs follow-up (separately in men and women)

For men, the only clinical variables that changed significantly over time were systolic and diastolic blood pressure, which reduced, and HbA_1c_, where a small increase was observed. Perceived behavioural control showed a small decrease, while significant improvements were seen for self-reported health status, anxiety, physical functioning and mental health. Men increased their physical activity (PAEE) from 8.2 to 9.1 kj/min over the year, but this change was not statistically significant. For women, small but significant increases were seen for BMI and HbA_1c_, with significant decreases in systolic and diastolic blood pressure over the same period. Women reported a small decrease in perceived behavioural control and intention, but a significant increase in health status and physical functioning. Increases in physical activity and fitness were observed, with the change in PAEE reaching statistical significance. Overall, the whole *ProActive *cohort (n = 321) significantly increased their PAEE by 0.6 kj/min (SD 4.2, t-statistic = 2.8, p = 0.006) over one year.

Table 2 shows the associations between demographic, clinical, psychosocial and behavioural variables at baseline and change in PAEE (kj/min) over one year. Male sex was associated with a significant increase in PAEE over one year, while age and social class did not demonstrate any association. Individuals with a higher level of fitness at baseline were more likely to increase their PAEE over one year. No significant associations were seen for any of the remaining clinical or psychosocial variables and PAEE. Weekly alcohol intake and baseline PAEE were positively associated with PAEE at follow-up. Each model explained 32 to 37% of the variance in one year PAEE change. However, baseline PAEE accounted for most of the variance in each model, with predictor variables typically adding less than 1% to the explained variance.

**Table 2:** Associations between baseline demographic, clinical, psychosocial and behavioural variables with change in PAEE (kj/min) over one year in the *ProActive *trial cohort, *n *= 321

Baseline variable	PAEE (kj/min) unstandardised b-coefficient (95% CI)^a^	P-value
*Demographic*		

Age (years)	-0.043 (-0.114 to 0.028)	0.233
Sex (female = 0; male = 1)	2.181 (1.286 to 3.075)	< 0.001
Social class (based on the IMD^b ^score 1-5; 5 representing highest social class)	0.283 (-0.204 to 0.771)	0.254

*Clinical*		

BMI (kg/m^2^)	0.019 (-0.068 to 0.105)	0.675
Systolic blood pressure (mm Hg)	0.000 (-0.032 to 0.031)	0.983
Diastolic blood pressure (mm Hg)	-0.016 (-0.060 to 0.029)	0.483
HbA_1c _(%)	-0.146 (-0.993 to 0.702)	0.736
Total cholesterol (mmol/l)	-0.204 (-0.633 to 0.225)	0.350
VO_2max _(l/min)	1.138 (0.644 to 1.631)	< 0.001

*Psychosocial*		

Perceived behavioural control (1 to 5)^c^	-0.086 (-0.795 to 0.623)	0.812
Intention (1 to 5)^c^	0.281(-0.390 to 0.953)	0.410
Current health status (scale 0-100, where 0 = worst imaginable health to 100 = best imaginable health state)	-0.019 (-0.047 to 0.008)	0.171
Anxiety score (Spielberger)	0.012 (-0.023 to 0.047)	0.494
SF-36 physical functioning score	-0.018 (-0.047 to 0.010)	0.208
SF-36 mental health score	-0.016 (-0.043 to 0.012)	0.275

*Behavioural*		

PAEE (kj/min)^d^	0.599 (0.506 to 0.691)	< 0.001
Self-reported physical activity index (met hours/week)	0.008 (0.000 to 0.016)	0.055
Self-reported alcohol (total units/wk)	0.059 (0.009 to 0.109)	0.022
Self-reported smoking status (0 = never/ex; 1 = current)	1.064 (-0.098 to 2.225)	0.072

^a ^Adjusted for baseline PAEE; ^b ^Index of multiple deprivation; ^c ^Theory of Planned Behaviour; ^d ^Unadjusted

After adjusting for all significant predictor variables in a multivariable regression model (baseline PAEE, sex, fitness, and self-reported alcohol consumption), baseline PAEE (β 0.454; 95%CI 0.349 to 0.560), sex (β 1.319; 95%CI 0.189 to 2.449) and fitness (β 0.668; 95%CI 0.064 to 1.273) remained as independent predictors of change in PAEE in this model. The variance explained by this model was 39%.

## Discussion

This study aimed to identify demographic, clinical, psychosocial and behavioural predictors of change in objectively measured physical activity over a one year period. Our findings suggest that very few of the variables measured at baseline in the *ProActive *trial cohort predicted change in PAEE over one year. Male sex and higher fitness levels were independently associated with significant increases in PAEE. Weekly alcohol intake was a significant predictor of change in PAEE but this association became non-significant when sex and fitness were included in the model. No significant associations with change were seen for any other variables. The finding that men were more likely than women to increase their physical activity over one year has been shown in some interventions [40] but not in others [12,41]. To the best of our knowledge, the result that individuals with higher fitness at baseline were more likely to increase their objectively measured physical activity is a novel finding.

### Comparison with other literature

In general, our results do not support the limited literature showing associations between certain socio-demographic, psychosocial and behavioural variables, and change in physical activity behaviour [14,42]. Younger age is usually associated with increased physical activity, both cross-sectionally and over time [20]. We did not show a significant relationship with age, though the range was quite restricted (30-50 years). Furthermore, most previous results come from observational studies rather than intervention studies, such as the *ProActive *trial, in which all participants were encouraged to increase their activity levels. Prospective studies that have examined the relationship between baseline predictors and level of physical activity at follow-up have demonstrated associations with self-efficacy [14,41,43-45]; perceived behavioural control [46]; past behaviour [43]; perceived benefits or views of exercise [45,47]; and the participant's belief that physical activity was important for their health [13]. However, the questions derived from the Theory of Planned Behaviour to characterize perceived behavioural control and intention about becoming more physically active over the next year did not predict *change *in physical activity in this cohort. These findings suggest that such measures may not be useful in identifying subgroups of participants who are more or less likely to make long-term changes in their activity levels. The positive association between fitness and change in PAEE is a novel finding. As fitness is partially genetically determined [48], for two people starting at the same physical activity level but with different fitness levels, it may be easier for the individual with a higher fitness level to increase their physical activity.

One of the challenges in comparing our results to those found in other studies is the difference in methods used to measure physical activity. Previous studies usually measured *current or future self-reported behaviour*, and frequently focused on sporting activities [15], while we examined *change in everyday objective physical activity behaviour*. When physical activity behaviour and physical activity determinants are both measured using the same method e.g. by self-report questionnaire, associations may be due, at least in part, to commonality in response patterns to these measures ("common method variance") [49]. Consequently, when physical activity is measured by a different method (e.g. heart rate monitoring), part of the correlation explained by common method variance disappears, producing lower, non-significant or non-existent associations. Indeed, studies using objectively measured behaviour are less likely to observe significant associations with predictors, such as those based on the theory of planned behaviour, than those using self-reported behaviour [22]. However, when we examined the relationship between the same predictors and change in self-reported physical activity, we found only a few significant associations (for male sex, and higher fitness and BMI at baseline), indicating that common method variance cannot fully explain the lack of association with objectively measured physical activity. A number of other reasons for the lack of association between baseline variables and physical activity change might also be considered.

### Potential reasons for lack of associations

First, previous work has focused on selected populations with a short follow-up [15]. We examined a sample of middle-aged, slightly overweight individuals, identified through primary care registers via a first degree family history of diabetes. This group represents a sub-group of the population who would benefit from increased overall physical activity. The longer than usual follow-up period in the *ProActive *trial (one year) may account for some of the differences found between this and other studies. Second, although our sample size was larger than many other physical activity promotion trials, there may have been a lack of heterogeneity in the cohort. This would have reduced our power to detect any associations with physical activity. However, the large standard deviations and ranges for change in PAEE indicate that this is unlikely to be the case.

Third, we did not measure some of the variables that have been shown to be associated with physical activity behaviour, including environmental and social factors, and may therefore have focused on the weaker determinants of objectively measured physical activity behaviour. King *et al*, for example, found that neighborhood environment is a potential predictor of physical activity maintenance over two years, though a self-report measure of physical activity was used [14]. Wider collective determinants of physical activity may act independently or interact with psychosocial correlates to influence physical activity behaviour, though these relationships have not been clearly elucidated [50,51]. Furthermore, some of the associations may have been weaker in our study because of the low reliability of the measures, e.g. Cronbach's alpha was only 0.54 for perceived behavioural control.

Fourth, since all participants were at risk due to their family history of disease, we cannot examine whether this factor was an important predictor of change. Similarly, all participants were subject to trial recruitment procedures and objective physical activity measurement (albeit without feedback), and the effects of this may have diluted associations reported in other studies. However, individuals in observational studies have also been recruited and measured, and these processes have been linked to behaviour change [52]. Objective measurement of physical activity in the *ProActive *cohort may have increased the saliency of this behaviour and prompted self-monitoring, an effective behaviour change technique in its own right.

Fifth, the precision in the methods we used to assess PAEE may also have contributed to the lack of observed associations. PAEE from individually calibrated heart rate monitoring is an integrated measure of energy expenditure above rest calculated from free-living heart rate data. In sedentary populations, much of the daytime is spent in the region around the flex heart rate, which is used to discriminate between rest and physical activity. The association between heart rate and energy expenditure is less precise in this region, which may reduce the accuracy of predicted PAEE on an individual level [53]. Finally, we conducted multiple significance tests (> 20) between baseline predictors and change in PAEE which may have led to an increased risk of Type 1 errors. It is unclear whether the few significant observations represent real or chance associations. The exploratory and post-hoc nature of our analyses mean that results should be interpreted with caution.

However, the *ProActive *trial allowed objective measurement of physical activity over a period of 12 months in a well-defined and accessible group of individuals who were at increased risk of diabetes and who would benefit from increases in physical activity. There was a high follow-up rate (88%). Objective measurement of physical activity reduces the error and bias commonly associated with self-report measures, and PAEE has been extensively validated in the laboratory and during free-living conditions [53,54]. Furthermore, we measured a wide range of potential predictors from a variety of domains, including clinical, behavioural, demographic and psychosocial variables.

### Future research implications

Systematic application of methods aimed at increasing our understanding of how predictors from different domains influence physical activity change over time remains scarce [42]. Yet, the potential benefit of examining these variables for physical activity, diet and weight control is well recognized in the behavioural and social sciences literature [55]. Identifying predictors of change in physical activity, regardless of whether the predictors are causally related to change, could help the targeting of appropriate sub-groups for intervention and improve efficacy. We could target those who were likely to change with brief interventions, such as action planning or goal setting in combination with self-monitoring, or target those who were unlikely to change with more intensive interventions [56]. Results from this analysis suggest that trying to predict who will change their physical activity behaviour is not straightforward. Ideally we would target people on the basis of both their risk of future health problems and the likelihood that they would change in response to intervention, but further research is needed to improve our understanding of determinants of change in physical activity behaviour. In addition, the lack of associations between traditional determinants of self-reported physical activity and behaviour change in this cohort suggests these factors may not be the most appropriate to inform intervention development.

## Conclusions

This study is one of largest to allow prospective examination of these variables with objective measurement of physical activity over one year. In contrast with previously described determinants of self-reported physical activity, very few baseline demographic, clinical, behavioural and psychosocial variables were associated with change in objectively measured physical activity over 12 months in the *ProActive *trial cohort. Our results suggest that predicting who will increase their physical activity is difficult, and that effective interventions may therefore need to be informed by other categories of determinants not measured here. Further research is needed to improve prediction of change in physical activity, and inform the development and targeting of interventions. As many of our prediction models were hypothesis-generating, our results will need validating in other experimental studies.

## Abbreviations

**EE**: Energy expenditure; **HbA_1c_**: Glycosylated haemoglobin; **HR**: Heart rate; **MREC**: Multi-centre Research Ethics Committee; **PAEE**: Physical activity energy expenditure; **REE**: Resting energy expenditure; **STAI**: Spielberger's State Anxiety Inventory;

## Competing interests

The authors declare that they have no competing interests.

## Authors' contributions

SJG conceived of the study question. RKS completed the analysis and drafted the manuscript. SJG, WH and SS participated in the study design and coordination. All authors contributed to analysis and interpretation of data, and read and approved the final manuscript.

## Pre-publication history

The pre-publication history for this paper can be accessed here:

http://www.biomedcentral.com/1471-2458/10/226/prepub
